# Work–Family Balance among Dual-Earner Couples in South Korea: A Latent Profile Analysis

**DOI:** 10.3390/ijerph18116129

**Published:** 2021-06-06

**Authors:** Sun-Young Ji, Hye-Sun Jung

**Affiliations:** 1 Department of Public Healthcare, Graduate School, The Catholic University of Korea, 222 Banpo-daero, Seocho-gu, Seoul 06591, Korea; ttresy@naver.com; 2 Department of Preventive Medicine, College of Medicine, The Catholic University of Korea, 222 Banpo-daero, Seocho-gu, Seoul 06591, Korea

**Keywords:** birth rate, South Korea, double-earner couple, work–family balance, latent profile analysis (LPA), typology

## Abstract

The declining birth rate in South Korea is concerning and linked to stress in the work–family balance, which is known to affect family planning. Therefore, providing proper support to double-earner couples might help improve the fertility rate. Work–family balance refers to the ability of individuals to perform their roles at work and home with equal involvement. This study identifies two aspects: gains and strains. Latent profile analysis is employed to create a typology that can account for the diversity in work–family balance. This approach is person-centered rather than variable-centered, and it identifies clusters of individuals that differ qualitatively, thereby examining the strains and gains experienced by double-earner couples. We classify the types of work–family balance and identify the attributes of each profile. The findings showed that men were more likely to belong to the high-gain class when they had a temporary position, multiple children, good health, low depression, higher life satisfaction levels, and strong social support. Women were more likely to belong to the high-gain class when they experienced high happiness levels and strong social support. Based on these profiles, we provide recommendations to enhance work–family balance, thereby contributing to strategies to overcome low birth rates.

## 1. Introduction

In 2018, the average total fertility rate of 37 OECD member countries was 1.63, of which South Korea had a birth rate of less than 1 (0.98) [1]. In 2019, Korea’s total fertility rate was 0.918, which is a record low in history [2]. Excluding regions with wars or famines, such a naturally low birth rate (that is, below 1.0) has occurred only in South Korea.

In 2020, a sharp population decline became a reality when the number of newborn babies was 20,000 fewer than the number of deaths. The decrease in total fertility rate is occurring at a concerning rate and far more rapidly than was forecasted. Although it is not likely that the low birth rate will rebound, it is imperative to slow down the rapid population change through a support policy for low birth rate and an aging society and taking measures to cope with the changes in population structure in a stable manner.

In 2005, the Korean government passed the *Framework Act on Low Birth Rate in an Aging Society*. Starting in 2006, the government has been implementing the Plan for Ageing Society and Population and renewing the plan every 5 years. Even though over 185 trillion KRW was invested in the plan until 2020, the birth rate has been steadily decreasing.

The early policies were centered around women and encouraged childbirth and child-care, because Korean women are generally more devoted to child-rearing than men [3]. Nonetheless, some scholars have pointed out that the policies were limited in their provision of a fundamental solution to an issue that originated in the social structure and that it was necessary to create paradigm shifts, such as the establishment of a policy for work–family balance [4,5].

In 2018, the number of double-earner households was 46.3% [6], and women’s participation rate in economic activities was 52.9% [7]. Both rates are gradually increasing each year. Double-earner couples have been reported to experience role stress while balancing work and family [8,9], and stress in the work–family balancing process affects the intention to give birth to a child [10]. In this vein, it is imperative to provide proper support for double-earner couples to improve the fertility rate.

Spillover models for work–family relationships have received the most attention both theoretically and empirically over recent decades. Individual emotions, attitudes, and behaviors obtained from one domain are considered to be shifted to other domains [11].

There are two aspects to work–family balance: gains and strains. In early studies, most scholars focused on work–family strains, which are often defined as an imbalance resulting in conflict and imbalance. In this study, work–family strain is a conflict between incompatible roles in the work–family domain [11].

Grenhaus and Beutel [12] identified time constraints, fatigue, burnout, and family adjustment as strain factors. Marshall and Barnett [11] reported that work–family strains are affected by the level of experience with work and parenting in terms of workload, the amount of time invested in child-rearing and household chores and caring for young children. The survey about the work–family interface among married couples with young children showed that men’s work–family strains increased when the number of hours of childcare or the number of preschool-aged children was high, while women’s work–family strains increased when there was a challenge in the child-rearing process or when they worked long hours [13]. In the meantime, the relationships among the variables may vary depending on the developmental stage of children [14,15].

Recent studies have focused on work–family gains. When there is a work–family balance, there are various benefits that are variously defined as work–family gains and include enrichment and balance. Work–family gain can, depending on the circumstances, lead to the expansion of resources, improvements between roles, and/or other welcomed outcomes [11,16]. In other words, work–family balance refers to a situation where individuals can perform the role of employees at work and the role of family members at home with equal involvement.

Greenhaus and Powell [16] argued that experience in different roles allows one to acquire interpersonal skills, coping skills, self-efficacy, and support intangible, psychological, and physical areas. Eventually, experience can be manifested in the form of higher energy. McNall et al. [17] argued that work–family balance improves emotional attachment to the organization, work–family satisfaction, and physical and mental health.

As such, strains and gains coexist in the work–family balance and should be studied simultaneously. However, previous studies examined only one aspect and showed limitations in that they could not analyze complicated situations in real life [18,19]. Moreover, most studies have focused primarily on women [20,21].

Work–family balance can manifest differently depending on individual characteristics in the following ways: high in both strains and gains, low in both strains and gains, and high in one aspect and low in the other aspect [22,23]. To account for such diversity in real life, this study employed latent profile analysis (LPA) for typology. LPA can successfully identify different clusters of individuals that differ qualitatively, and can compensate for the limitation by taking a person-centered approach as opposed to a variable-centered approach, and comprehensively examine the strains and gains of double-earner couples.

Additionally, the study sought to identify differences in relevant predictors to provide information about the factors that determine work–family balance patterns. The interaction variables related to work–family balance are diverse, and predictors were selected based on previous studies. The demographic and sociological variables include age, educational background, job type, income, and number of children [24].

The literature on self-assessed health and work–family balance continues to grow [25]. Beutell and Wittig-Berman [26] considered differences in behavioral health habits such as smoking and drinking. Psychosocial support can buffer work–family stress. Life satisfaction is often evaluated as an outcome variable when there is sufficient work–family balance, and there are studies on the interaction between work–family imbalance and depression [27,28].

In this study, we classify the double-earner couples’ types of work–family balance and identify the attributes of each profile. Based on the analysis of the characteristics, we can provide implications for the establishment of a preventive intervention program for each profile. In doing so, this study aims to contribute to establishing strategies to overcome low birth rates.

## 2. Materials and Methods

### 2.1. Sample and Data Collection

For this study, we analyzed survey data from the 10th Panel Study on Korean Children [29]. The PSKC is conducted every year, targeting a panel household of 2150 children born in South Korea in 2008 by the Korea Institute of Child Care (KICCE) using a stratified multi-stage sampling method.

We used data from the 10th survey (2017) when the panel of children were 10 years old. From the original responses of 1484 households in the 10th survey, 1304 dual-income couples were selected: 1299 fathers and 751 mothers who responded to the questions related to work–family balance as the final targets of the analysis.

### 2.2. Measurements

#### 2.2.1. Work–Family Balance 

To measure work–family balance, we used the *work–family strains and gains scales* developed by Marchall and Barnett [30]. Specifically, we used the questions that KICCE translated from the original scales for the panel study. Marshall and Barnett [11] argued that family life and parenting should be examined in detail because the quality of experience in the job and parenting roles contributes to work–family strains. Based on this, Marchall and Barnett [30] divided *work–family* and *work–parenting* and created four subcategories with a total of 26 questions. Each item was measured on a 5-point Likert scale, ranging from *strongly disagree* (1 point) to *strongly agree* (5 points). Higher scores imply a stronger impact for each factor. In this study, we used the mean of the sum of each sub-factor This study Cronbach’s α 0.777. Examples of subfactor questions are as follows:

- Factor I. Work–family gains: This sub-factor consists of seven questions and measures the positive aspect of work–family balance. Higher scores indicate a greater perception of gains in work–family balance. An example of a question in this category includes, “taking responsibility at work–family makes me a more balanced person.” This sub-factor Cronbach’s α 0.927.
- Factor II. Work–family strains: This sub-factor consists of nine questions and measures the negative aspects of work–family balance. Higher scores indicate a greater perception of strain in work–family balance. An example of a question in this category includes “things to do at work interfere with time spent with family.” This sub-factor Cronbach’s α 0.868.
- Factor III. Work–parenting gains: This sub-factor consists of four questions and measures the positive aspects of work–parenting balance. Higher scores indicate a greater perception of gains in work–parenting balance. An example of a question in this category is “my work for has a positive effect on my child.” This sub-factor Cronbach’s α 0.869.
- Factor IV. Work–parenting strains: This sub-factor consists of six questions and measures the negative aspects of work–parenting balance. Higher scores indicate a greater perception of strain in the work–parenting balance. An example of a question in this category is “my job seems to put a strain on the child.” This sub-factor Cronbach’s α 0.847.

#### 2.2.2. Demographic Characteristics

To identify the types of work–family and work–parenting gains and strains, we selected the following characteristics as variables: demographic characteristics, household and couple characteristics, health characteristics, and psychosocial characteristics.

For demographic characteristics, the following variables were used: age (≤39, 40–49, ≥50); educational level (≤high school graduate, college, university, or masters); employment status (Permanent, Self-Employed, Temporary).

#### 2.2.3. Household and Couple Characteristics

Characteristics of households and couples included: the number of children, household income (≤3.99 million, 4.00–4.99 million, 5.00–5.99 million, ≥6.00 million), and area (Urban, Rural, Suburban).

#### 2.2.4. Health Characteristics

Health characteristics included: alcohol use frequency (no, ≤once a week, ≥twice a week), smoking (never, ≤10, 11–20, ≥21), and subjective health status (score 1, very un-healthy; score 5, very healthy).

#### 2.2.5. Psychosocial Characteristics

Psychosocial characteristics included: depression (score ≥ 19: severe, score 14–18: mild, score ≤ 13: normal), subjective happiness (score 1 Unhappiness ~ score 7 happiness), daily stress (score 1 not at all, score 3 very much), life satisfaction (satisfied, neutral, dissatisfied), and social support (score 1: no support ~ score 5: high level of support). Each of these characteristics were measured using the mechanisms explained below:

- Depression (K6)

K6 consists of six questions that ask respondents how frequently in the past 30 days they had felt: (1) nervous, (2) hopeless, (3) restless or fidgety, (4) so depressed that nothing could cheer them up (depressed), (5) that everything was an effort, and (6) worthless. For each of these questions, the K6 included five response options: “never,” “a little of the time,” “some of the time,” “most of the time,” and “all of the time.” Responses were scored from 1 (“never”) to 5 (“all of the time”), score ≥19: severe, score 14–18: mild, and score ≤ 13: normal [31];

- Subjective happiness Scale (SHS)

The SHS consists of four questions that ask respondents to select the level that feels most appropriate to their life. 1) In general, I consider myself: (1) not a very happy person–(7) a very happy person. 2) Compared to most of my peers, I consider myself: (1) less happy–(7) more happy. 3) Some people are generally very happy. They enjoy life regardless of what is going on, getting the most out of everything. To what extent does this characterization describe you? (1) not at all–(7) a great deal. 4) Some people are generally not very happy. Although they are not depressed, they never seem as happy as they might be. To what extend does this characterization describe you? (1) not at all–(7) a great deal. The evaluation was made using the average of the four questions; responses were scored from 1 (unhappiness) to 7 (happiness) [32];

- Daily stress

The questions ask respondents about how much stress they usually experienced. For these questions, response options included: (1) not at all; (2) little; (3) very much. The evaluation was made using the average;

- Life satisfaction

These questions asked respondents how satisfied there currently are with their life. For these questions, response options included: “satisfied”, “neutral,” and “dissatisfied.”;

- Social support

This measures how much social support the family receives from outside the household by measuring four sub-factors: emotional, instrumental, informational, social network. This section consists of 13 questions.

Examples of subfactor questions are as follows:

(1) Emotional support: Always care about my work and worry about it;
(2) Instrumental support: Support the necessary item;
(3) Informational support: Information necessary for parenting children is available;
(4) Friendly support: Contact and visit frequently.

Responses were scored from 1 (“no support”) to 5 (“high level of support”), the evaluation was made using the average [33].

### 2.3. Statistical Analysis

This study used the SPSS 23.0 (IBM Corp, New York, NY, USA), and M-plus 8.0 (Muthen & Muthen, Louisiana, LA, USA) for analysis. The characteristics of the study participants were analyzed using frequency analysis and descriptive statistical analysis.

To derive the work–family balance types, we used latent profile analysis (LPA). Along with LPA, latent class analysis (LCA) is a method of identifying subgroups of individuals with similar characteristics based on the pattern of their responses to the observed variables. The potential group obtained through the LPA is called the “latent profile.” In the analysis process, the number of potential profiles for respondents is determined through the model verification value.

In this study, to derive the optimal number of potential groups, the number of potential groups was increased step by step, where each step increase was analyzed to understand the fit of each model, and an exploratory process was undertaken to find the optimal model. According to Muthen and Muthen [34], (1) Akaike information criterion (AIC) in which the smaller AIC value indicates a better fit model; (2) Bayesian information criterion (BIC) in which the smaller BIC value indicates a better fit model; (3) adjusted Bayesian information criterion (aBIC), in which the smaller aBIC value indicates a better fit model; (4) The bootstrap likelihood ratio test (BLRT) is a method of comparing the (k-1) class model with the (k) class model when the number of latent classes is k. When the value is statistically significant (*p* < 0.05), the (k-1) class model is rejected, and the (k) class is adequate; (5) Entropy is a fit index that shows the accuracy of the model’s categorization. The entropy indices range from 0 to 1, and a value closer to 1 indicates accurate categorization. As for the sample size, we chose 5% because Jung and Wickrama [35] claimed that a sample size of over 5% of the total samples is appropriate for comparing classes.

To examine the relationship between each characteristic and the types of work–family strains and gains derived through latent profile analysis, we conducted a chi-squared analysis. To verify the influence of related variables for each type, we performed a multinomial logistic regression analysis.

### 2.4. Ethical Considerations

All versions of the PSKC were executed by the KICCE, and all subjects provided written informed consent before participating in the survey.

The protocol of the present study was approved by the Institutional Review Board of the Catholic University (MIRB-MYUN20201023-002; approval date: October 23, 2020) and conducted following the rules of the Declaration of Helsinki.

## 3. Results

### 3.1. Sample Description

The sample in this study was comprised of 1299 men (87.5%) and 751 women (50.6%) who responded to questions about work–family balance. The average age of the participants was 42 and 39 for men and women, respectively. Concerning educational level, college graduates constituted the majority (41% for men and 39.4% for women). Regarding employment status, a vast majority of the participants held a permanent position (79.5% for men and 65.5% for women), followed by self-employment (15.9% for men, 22.3% for women) and temporary positions (4.5% for men and 12.2% for women). The average number of children was two. The average monthly household income was 5,320,000 KRW for men and 5,790,000 KRW for women. Most participants were non-smokers (55.0% for men and 97.9% for women). Regarding alcohol use, most participants consumed alcohol once or more per week (49% for men and 64.6% for women). Concerning subjective health conditions, most participants perceived their health condition to be good or fair (3.39 points for men and 3.41 points for women). Regarding depression levels, most participants showed a normal range (67.6% for men and 69.7% for women), followed by mild depression (24.5% for men, 21.8% for women), and then severe depression (7.8% for men and 8.5% for women). In this study, we also observed groups with relatively high levels of depression. The subjective happiness level was 5.2 points, which is an above-average level. Regarding daily stress levels, most participants reported that they experienced stress to a small degree (2.04 points for men, 1.95 points for women). Most participants also showed satisfaction with life (54.0% for men and 55.0% for women). Concerning social support, the participants received above-average levels of support (3.94 points for men; 3.91 points for women). This information is laid out fully in Table 1.

### 3.2. Latent Profile Analysis

#### Fit Indices for the Latent Profile Models on Work–Family Balance

The fit indices for the latent profile models on men’s work–family balance (Table 2), indicated that AIC, BIC, and SABIC decreased as the number of latent classes increased. The fit indices of the model with four latent classes were found to be the lowest. The entropy of the model with four latent classes was 0.784, which is higher than that of the other latent class models. In BLRT, the models with two or three latent classes were significant at *p* < 0.001. The model with four latent classes was found to have less than 5% latent classes. When comprehensively considering the fit indices for the latent profile model, we selected the model with three latent classes as the final model, as it was deemed most adequate for work–family balance classification.

**Figure 1:**
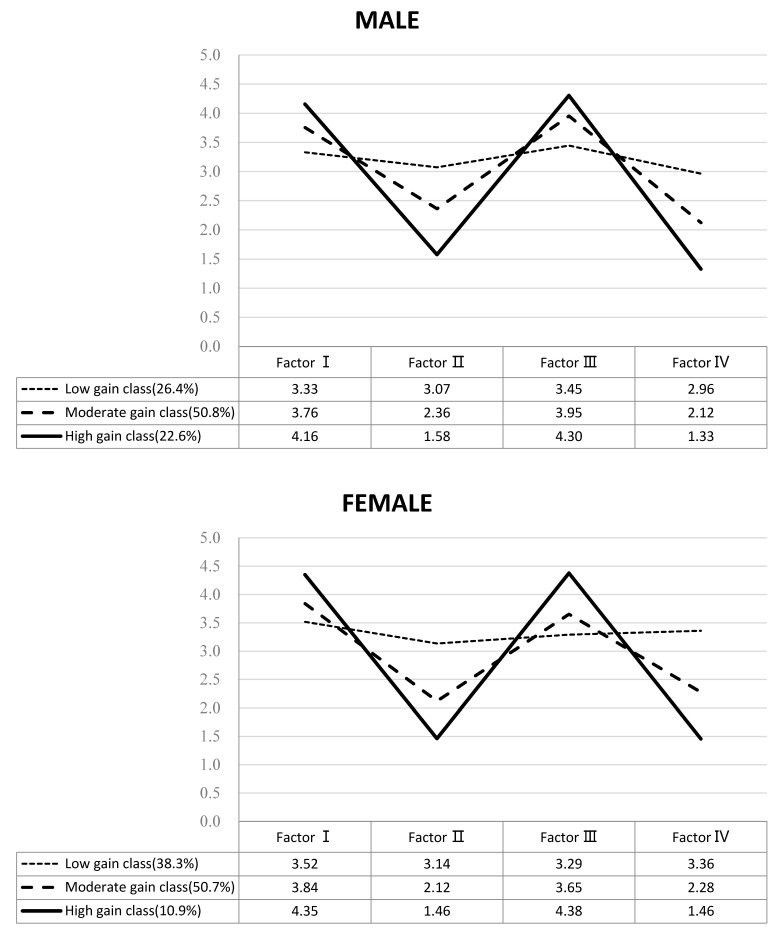
Pattern of work–family balance scores.

Male work–family balance scores (Figure 1) showed three types:

- Class 1 (344 cases, 26.4%) was labeled as the *low gain class*. The average scores of gains and strains were close to 3.2 points, respectively, and manifested the characteristics of low gains and highest strains as follows: 3.3 points in work–family gains, 3.4 points in work–parenting gains, 3.1 points in work–family strains, and 3.0 points in work–parenting strains;
- Class 2 (661 cases, 50.8%) was labeled as the *moderate gain class*. The average scores of gains and strains were 3.9 points and 2.3 points, respectively, and manifested the characteristics of middle gains and lower strains as follows: 3.8 points in work–family gains, 4.0 points in work–parenting gains, 2.4 points in work–family strains, and 2.1 points in work–parenting strains;
- Class 3 (294 cases, 22.6%) was labeled as the *high gain class*. The average scores of gains and strains were 4.3 points and 1.5 points, respectively, and manifested the characteristics of highest gains and lowest strains as follows: 4.2 points in work–family gains, 4.3 points in work–parenting gains, 1.6 points in work–family strains, and 1.3 points in work–parenting strains.

When comprehensively considering the fit indices for the latent profile models on women’s work–family balance (Table 2), we selected the model with three latent classes as the final model, as it was deemed most adequate for work–family balance classification.

Female work–family balance scores (Figure 1) also showed three types. Based on the characteristics, the types were labeled as follows: Class 1 (288 cases, 38.3%) as *low gain class*; Class 2 (381 cases, 50.7%) as *moderate gain class*; and Class 3 (82 cases, 10.9%) as *high gain class*.

The latent profile model of work–family balance showed that double-earner couples perceived more gains than strains. None of the latent profile types of work–family balance were negative, meaning that it only manifested a high level of strain.

### 3.3. Differences in Characteristics According to Work–Family Balance Pattern

To examine the work–family balance patterns more closely, we analyzed the differences in characteristics such as demographics, household and couple characteristics, health, and psychosocial characteristics (Table 3).

In comparison with the *low gain class*, men in the *high gain class* scored higher in the following variables: age (𝜒 ^2^ = 11.51, *p* < 0.05), self-employment, temporary position (𝜒 ^2^ = 13.49, *p* < 0.01), non-smoker (𝜒 ^2^ = 18.38, *p* < 0.01), normal range of depression (𝜒 ^2^ = 182.49, *p* < 0.001), life satisfaction (𝜒 ^2^ = 141.27, *p* < 0.001).

In comparison with the *low gain class*, women in the *high gain class* scored higher in the following variables: normal range of depression (𝜒 ^2^ = 33.13, *p* < 0.001) and life satisfaction (𝜒 ^2^ = 35.10, *p* < 0.001).

### 3.4. Multinomial Logistic Regression Analysis

We conducted a multinomial logistic regression analysis to investigate how work–family balance patterns are influenced by demographic, household and couple, health, and psychosocial characteristics. To identify the characteristics of the high-gain class whose work–family gains were high, we verified the relationship with the variables using the low-gain class pattern as a reference.

We examined the characteristics of men who were likely to belong to the moderate-gain class as opposed to the low-gain class (Table 4). The result showed that the likelihood increased by 2.77 times for men with a temporary position as opposed to a permanent position (95% CI, 1.01–7.61) and 2.05 times for men with better life satisfaction (95% CI, 1.08–3.89). In contrast, men with depression were less likely to belong to the *moderate gain class* (0.36 times for men with severe depression, 0.40 times for men with mild depression).

We examined the characteristics of men who are likely to belong to the high-gain class as opposed to the low-gain class. The results showed that the likelihood of belonging to the high-gain class increased by 1.72 times for men with multiple children (95% CI, 1.22–2.42), and 1.57 times for men with good subjective health (95% CI, 1.11–2.22). Moreover, the likelihood of belonging to the high-gain class increased by 2.58 times for men with a higher level of subjective happiness (95% CI, 1.84–3.62) and 1.49 times for men with stronger social support (95% CI, 1.02–2.17). In contrast, the likelihood of belonging to the high-gain class decreased by 0.43 times for men with a higher level of daily stress (95% CI, 0.29–0.64), and for men with depression (0.37 times for severe depression, 0.28 times for mild depression).

We examined the characteristics of women who were likely to belong to the high-gain class as opposed to the low-gain class (Table 5). The results showed that the likelihood for women belonging to the high-gain class increased by 2.06 times for women with a high level of subjective happiness (95% CI, 1.33–3.20) and 6.08 times for women with stronger social support (95% CI, 3.42–10.82).

## 4. Discussion

### 4.1. Main Findings

This study aimed to categorize the level of work–family balance in double-earner couples and to identify the different effects of characteristics on each pattern.

We categorized the patterns of work–family balance in double-earner couples into three classes for both men and women: low gain, moderate gain, and high gain. Whereas Lee and Gu [22] categorized the patterns of work–family interfaces for working mothers with preschool-aged children into four classes, Kim et al. [23] employed cluster analysis and categorized the patterns of work–family balance in working fathers. The present study is significant in that it overcame the limitation of cluster analysis by employing latent profile analysis and it included double-earner couples in the work–family balance patterns.

In this study, the double-earner couples perceived more gains than strains. None of the work–family balance patterns were of a negative type that only manifested a high level of strain. This finding matches the findings from previous studies showing that there were gain-dominant classes in work–family balance patterns [22,23]. It also supports the expansionist theory that performing multiple roles is good for health [36,37].

The subjects of this study may have managed the conflicts well compared with parents whose children were younger because their level of family adjustment was higher as parents of 11-year-old children. Moreover, the subjects may have perceived gains because they were well-educated and earning above-average household incomes.

Nevertheless, the level of work–family balance needs continual monitoring and preemptive intervention by taking the accumulative impact into account, because long-term changes may occur. Particularly for the *low gain class*, who manifest little difference between gains and strains, it is essential to provide a focused-intervention because they are the class most at risk among all work–family balance types.

We examined the factors that determine the patterns of work–family balance. First, when a man held a temporary as opposed to a permanent position, the likelihood of belonging to the *high gain class* increased by a factor of 2.77. Similarly, Yoon et al. [38] targeting married men, showed that men working as employers or self-employed as opposed to permanent employees perceived their work–family life to be balanced. These findings indicate that the working environment can be a fundamental factor in work–family balance.

Despite the desire for work–family balance, long work hours can limit the time distribution [39,40], and particularly, double-earner couples can perceive greater strain due to their childcare responsibilities. For double-earner couples to secure enough time for their family, it is essential to establish a policy to reinforce flexibility at work, such as maternity leave, parental leave, and flexible work hours. As the data used in the present study did not include information on occupation and work hours, we could not compare the details. In a follow-up study, however, it will be necessary to identify the influencing factors by reflecting the various characteristics of the working environment.

Second, when a man had multiple children, the likelihood of belonging to the high-gain class increased by a factor of 1.72. In women, the number of children was not significant. While men with multiple children perceive a higher level of work–family gains from psychological support from their spouse and children [41], women with multiple children or young children tend to perceive a higher level of work–family strains [42,43].

As women feel the greater burden of child-rearing, men’s gender role attitude may not be significantly affected by the number of children [44]. In this light, it is necessary to redefine the double-earner couple’s roles in sharing household chores and child-rearing responsibilities and to provide support to increase work–family gains by vitalizing a mandatory, family-friendly system. Moreover, it is necessary to conduct a follow-up study to investigate diverse family variables based on children’s developmental stages because the level of strain can vary based on the children’s ages.

Third, when a man perceived his health to be in good condition, the likelihood of belonging to the high-gain class increased by a factor of 1.57. Work–family balance has been reported to affect the health of the body and mind [45,46]. It was also found that men who believe that they are healthy are more likely to perceive their work–family life as balanced [47]. Therefore, it is necessary to provide health improvement programs for double-earner couples and create an environment that encourages participation in the programs. In most studies, personal health conditions were found to have a close relationship with work–family balance. However, the present study showed that the health factor was significant only for men. Therefore, a follow-up study is necessary to verify the differences in effect based on gender.

Fourth, when a man had a lower level of depression and stress and a higher level of life satisfaction and happiness, the likelihood of belonging to the high-gain class increased. Subjective happiness was also a significant factor in women. Many previous studies have shown that depression and role conflict are the main causes of work–family strains [27,28]. Some scholars advocate promoting the positive aspect of work–family life by increasing the level of satisfaction [48].

When working couples perceive performing multiple roles as helpful for everyone in their family, their level of satisfaction and happiness tends to increase, and they are more likely to successfully perform the role of parent or spouse. In other words, it is crucial to increase work–family gains not only through support for work–family strains but also through mental and psychological support. In this vein, it is necessary to provide programs that can promote the happiness of double-earner couples and manage their stress and depression while monitoring psychological changes.

Lastly, when working couples had stronger social support, their likelihood of belonging to the *high gain class* increased by a factor of 1.49 for men and 6.08 for women, respectively. For women, social support is a key factor for determining the patterns of work–family balance. Many studies have also addressed the importance of social support for work–family balance [49,50,51]. Kim and Yang [52] argued that it is imperative to first increase awareness and create a suitable atmosphere for the implementation of policies to provide social support.

The patterns of work–family balance can be manifested in a complex manner depending on family interaction and the utilization of available resources, as opposed to personal behaviors or circumstances. Furthermore, a lack of perceived conflict can probably be interpreted as a lack of actual conflict [53]. Therefore, it is necessary to create an environment for double-earner couples to receive sufficient social support, check the required resources, and improve the effectiveness of resource utilization.

### 4.2. Strengths and Limitations

In this study, we used the 10th survey data from 2017 to check the current position. We also derived more realistic research results by taking a person-centered approach as opposed to a variable-centered approach to assessing the work–family balance of double-earner couples. The limitation of this study is that we used secondary data and could not set up diverse factors that determine the patterns of work–family balance. Nonetheless, the findings of this study can be generalized because the data used in this study were collected from a nationwide survey, which is a strength of this study. In follow-up research, it will be necessary to explore the relationship between variables including the family variables and the characteristics of the working environment for different groups of double-earner couples based on children’s developmental stages.

It is difficult to compare this study’s findings with those of previous studies because few scholars have researched the same topic with the same set of variables using an identical method of analysis. At present, when work–family balance policy has become a social issue as and a means to address the problem of a *low birth rate and aging society*, this study has significance because it widely investigated the patterns of work–family balance in double-earner couples in terms of strains and gains. The findings of this study provide information about factors determining the patterns of work–family balance, and can be used as baseline data for the development of policies for different patterns of work–family balance.

## 5. Conclusions

To contribute to the establishment of strategies to overcome the low birth rate in South Korea, we categorized the work–family balance in double-earner couples and identified the characteristics of each pattern.

We derived three patterns of work–family balance in double-earner couples. The perceived level of work–family gains was greater than that of work–family strains. However, it is necessary to continually monitor and preemptively intervene, because work–family balance may change in pattern from a long-term perspective, and not all patterns are the same.

The results indicate that the factors that determine the patterns of work–family balance were as follows: Men were more likely to belong to the *high gain class* when they had a temporary position as opposed to a permanent position, multiple children, good health, a low level of depression and stress, a higher level of life satisfaction and happiness, and strong social support. By contrast, women were more likely to belong to the high-gain class when they had a high level of happiness and strong social support. Social support is a key factor for women.

For double-earner couples to balance work and family, the following measures should be taken. First, flexible working conditions should be reinforced. Second, it is imperative to create an environment that improves the physical and mental health of double-earning couples so that they can function better at work and home. Third, it is crucial to increase work–family gains by providing support for work–family strains as well as mental and psychological support. To do so, managing stress and depression, providing programs to promote happiness, and monitoring changes in psychological conditions can be implemented. Fourth, double-earner couples should be offered access to sufficient social support by increasing the support resources with higher demand and improving the effectiveness of their resource utilization.

In this study, we examined various patterns of work–family balance in double-earner couples in terms of strains and gains. The findings of this study can contribute to the design of strategies to promote person-centered work–family gains.

## Figures and Tables

**Table 1:** General characteristics of respondents by gender.

Variable		Male (N = 1299)		Female (N = 751)	
		N (%)	M ± SD	N	M ± SD
**Demographic** **characteristic**	**Age (years)**		42.20 ± 3.884		39.95 ± 3.621
	≤39	319 (24.6)		361 (48.6)	
	40–49	926 (71.5)		377 (50.7)	
	≥50	50 (3.9)		5 (0.7)	
	**Educational level**				
	≤High school graduate	344 (26.6)		191 (25.4)	
	College	277 (21.4)		199 (26.5)	
	University	531 (41)		296 (39.4)	
	≥Masters	143 (11)		65 (8.7)	
	**Employment status**				
	Permanent	754 (79.5)		471 (65.5)	
	Self-employed	151 (15.9)		160 (22.3)	
	Temporary	43 (4.5)		88 (12.2)	
**Household and couple** **characteristics**	**Number of children**		2.21 ± 0.678		2.20 ± 0.667
	**Household Income (KRW/month)**				
	Less than 3.99 million	315 (27.0)	532.98 ± 447.456	126 (17.9)	579.41 ± 434.823
	4.00–4.99 million	237 (20.3)		128 (18.2)	
	5.00–5.99 million	278 (23.8)		167 (23.8)	
	Higher than 6.00 million	338 (28.9)		282 (40.1)	
	**Area**				
	Urban	510 (39.3)		303 (40.3)	
	Rural	75 (5.8)		44 (5.9)	
	Suburban	714 (55.0)		404 (53.8)	
**Health** **characteristics**	**Alcohol use frequency**				
	No	113 (8.7)		142 (20.0)	
	Less than once a week	636 (49.0)		458 (64.6)	
	More than twice a week	548 (42.3)		109 (15.4)	
	**Smoking (cigarettes/day)**				
	Never smoke	714 (55.0)		691 (97.9)	
	≤10	216 (16.6)		10 (1.4)	
	11–20	290 (22.3)		4 (0.6)	
	≥21	79 (6.1)		1 (0.1)	
	**Subjective health status**		3.39 ± 0.743		3.41 ± 0.760
**Psychosocial** **characteristics**	**Depression**				
	Severe	101 (7.8)	11.93 ± 4.526	60 (8.5)	
	Mild	318 (24.5)		154 (21.8)	
	Normal	878 (67.6)		493 (69.7)	
	**Subjective happiness**		5.20 ± 0.967		5.20 ± 1.041
	**Daily stress**		2.04 ± 0.625		1.95 ± 0.620
	**Life satisfaction**				
	Satisfied	701 (54.0)		392 (55.4)	
	Neutral	454 (35.0)		236 (33.3)	
	Dissatisfied	143 (11.0)		80 (11.3)	
	**Social support**		3.94 ± 0.593		3.91 ± 0.597

M = mean; SD = standard deviation.

**Table 2:** Fit indices for the latent profile models on work–family balance.

	Classes	AIC	BIC	SSABIC	Entropy	BLRT (*p*-Value)	Class Size (%)
**Male** **(*n* = 1299)**	1	10469.908	10511.263	10485.851	-	-	-
	2	9487.807	9555.009	9513.714	0.728	<0.001	682 (52.5), 617 (47.4)
	3	9200.4	9293.448	9236.271	0.738	<0.001	344 (26.4), 661 (50.8), 294 (22.6)
	4	9030.777	9149.672	9076.612	0.784	0.002	354 (27.2), 43 (3.3), 244 (18.7), 658 (50.6)
	**Classes**	**AIC**	**BIC**	**SSABIC**	**Entropy**	**BLRT** **(*p*-Value)**	**Class Size (%)**
**Female** **(*n* = 751)**	1	6274.821	6311.792	6286.821	-	-	-
	2	5635.035	5695.114	5653.834	0.807	<0.001	423 (56.3), 328 (43.6)
	3	5614.746	5597.932	5540.774	0.788	<0.001	288 (38.3), 381 (50.7), 82 (10.9)
	4	5413.305	5519.597	5446.563	0.831	0.001	13 (1.7), 372 (49.5), 81 (10.7), 285 (37.9)

AIC = Akaike information criterion; BIC = Bayesian information criterion; SSABIC = Sample size adjusted Bayesian information criterion; BLRT: bootstrapped likelihood ratio test.

**Table 3:** Differences in general characteristics according to work–family balance pattern.

Variable		Male (*n* = 1299)				Female (*n* = 51)			
		Low Gain Class	Moderate Gain Class	High Gain Class	X^2^	Low Gain Class	Moderate Gain Class	High Gain Class	X^2^
**Demographic** **characteristics**	**Age (years)**								
	≤39	91 (26.6)	146 (22.2)	82 (27.9)	11.51 *	133 (47.0)	181 (47.9)	47 (57.3)	6.45
	40–49	238 (69.6)	494 (75.0)	194 (66.0)		146 (51.6)	196 (51.9)	35 (42.7)	
	≥50	13 (3.8)	19 (2.9)	18 (6.1)		4 (1.4)	1 (0.3)	0 (0)	
	**Educational level**								
	≤High school graduate	107 (31.2)	161 (24.4)	76 (25.9)	9.15	85 (29.5)	90 (23.6)	16 (19.5)	9.86
	college	77 (22.4)	140 (21.2)	60 (20.5)		62 (21.5)	111 (29.1)	26 (31.7)	
	University	131 (38.2)	281 (42.6)	119 (40.6)		111 (38.5)	151 (39.6)	34 (41.5)	
	≥Masters	28 (8.2)	77 (11.7)	38 (13.0)		30 (10.4)	29 (7.6)	6 (7.3)	
	**Employment status**								
	Permanent	190 (78.2)	397 (80.9)	167 (78.0)	13.49 **	193 (69.7)	228 (63.0)	50 (62.5)	6.89
	Self-employed	47 (19.3)	63 (12.8)	41 (19.2)		49 (17.7)	88 (24.3)	23 (28.8)	
	Temporary	6 (2.5)	31 (6.3)	6 (2.8)		35 (12.6)	46 (12.7)	7 (8.8)	
**Household and couple** **characteristics**	**Household Income** **(KRW/month)**								
	Less than 3.99 million	86 (28.5)	155 (25.8)	74 (27.8)	3.23	50 (18.5)	64 (18.1)	12 (15.2)	1.89
	4.00–4.99 million	67 (22.2)	118 (19.7)	52 (19.5)		48 (17.8)	62 (17.5)	18 (22.8)	
	5.00–5.99 million	63 (20.9)	148 (24.7)	67 (25.2)		62 (23.0)	88 (24.9)	17 (21.5)	
	Higher than 6.00 million	86 (28.5)	179 (29.8)	73 (27.4)		110 (40.7)	140 (39.5)	32 (40.5)	
	**Area**								
	Urban	132 (38.4)	258 (39.0)	120 (40.8)	8.57	129 (44.8)	144 (37.8)	30 (36.6)	6.47
	Rural	13 (3.8)	50 (7.6)	12 (4.1)		11 (3.8)	26 (6.8)	7 (8.5)	
	Suburban	199 (57.8)	353 (53.4)	162 (55.1)		148 (51.4)	211 (55.4)	45 (54.9)	
**Health** **characteristics**	**Alcohol use frequency**								
	No	22 (6.4)	57 (8.6)	34 (11.6)	5.96	53 (20.2)	77 (20.9)	12 (15.4)	6.87
	Less than once a week	168 (48.8)	325 (49.2)	143 (48.8)		169 (64.5)	229 (62.1)	60 (76.9)	
	More than twice a week	154 (44.8)	278 (42.1)	116 (39.6)		40 (15.3)	63 (17.1)	6 (7.7)	
	**Smoking (cigarettes/day)**								
	Never smokers	163 (47.4)	378 (57.2)	173 (58.8)	18.38 **	254 (96.9)	361 (98.4)	76 (98.7)	11.60
	≤10	64 (18.6)	104 (15.7)	48 (16.3)		6 (2.3)	4 (1.1)	0 (0)	
	11–20	83 (24.1)	146 (22.1)	61 (20.7)		2 (0.8)	2 (0.5)	0 (0)	
	≥21	34 (9.9)	33 (5.0)	12 (4.1)		0 (0)	0 (0)	1 (1.3)	
**Psychosocial characteristics**	**Depression**								
	Severe	60 (17.4)	35 (5.3)	6 (2.0)	182.49 ***	35 (13.5)	23 (6.2)	2 (2.6)	33.13 ***
	Mild	142 (41.3)	148 (22.5)	28 (9.5)		76 (29.2)	66 (17.9)	12 (15.4)	
	Normal	142 (41.3)	476 (72.2)	260 (88.4)		149 (57.3)	280 (75.9)	64 (82.1)	
	**Life satisfaction**								
	Satisfied	101 (29.4)	389 (58.9)	211 (71.8)	141.27 ***	110 (42.1)	225 (61.0)	57 (73.1)	35.10 ***
	Neutral	168 (48.8)	217 (32.9)	69 (23.5)		108 (41.4)	110 (29.8)	18 (23.1)	
	Dissatisfied	75 (21.8)	54 (8.2)	14 (4.8)		43 (16.5)	34 (9.2)	3 (3.8)	

* *p* < 0.05, ** *p* < 0.01, *** *p* < 0.001.

**Table 4:** Multinomial logistic regression of characteristics according to male work–family balance pattern.

Variable		Male (*n* = 1299)					
		Class 2: Moderate Gain Class			Class 3: High Gain Class		
		*p*	OR	95% CI	*p*	OR	95% CI
**Demographic** **characteristics**	**Age (years)**						
	≤39	0.600	0.75	0.25–2.24	0.265	0.48	0.13–1.74
	40–49	0.748	1.19	0.41–3.47	0.314	0.53	0.15–1.84
	≥50	-	Ref.	-			
	**Employment status**						
	Temporary	0.048	2.77	1.01–7.61	0.801	1.19	0.31–4.52
	Self-employed	0.229	0.75	0.47–1.20	0.790	0.83	0.55–1.76
	Permanent		Ref.				
**Household and** **couple characteristics**	**Child number**	0.247	1.18	0.89–1.55	0.002	1.72	1.22–2.42
**Health** **characteristics**	**Smoking (cigarettes/day)**						
	Never smoke	0.968	1.02	0.47–2.21	0.927	0.95	0.32–2.81
	≤10	0.681	0.84	0.36–1.94	0.858	0.90	0.28–2.85
	11–20	0.806	1.11	0.49–2.48	0.903	0.93	0.30–2.90
	≥21		Ref.				
	**Subjective health status**	0.532	1.09	0.83–1.42	0.010	1.57	1.11–2.22
**Psychosocial characteristics**	**Depression**						
	Severe	0.002	0.36	0.19–0.68	0.090	0.37	0.12–1.17
	Mild	<0.001	0.40	0.27–0.60	<0.001	0.28	0.15–0.52
	Normal		Ref.				
	**Subjective happiness**	0.144	1.21	0.94–1.58	<0.001	2.58	1.84–3.62
	**Daily stress**	0.398	0.87	0.64–1.20	<0.001	0.43	0.29–0.64
	**Life satisfaction**						
	Satisfied	0.027	2.05	1.08–3.89	0.347	0.65	0.27–1.59
	Neutral	0.209	1.41	0.82–2.43	0.324	0.66	0.29–1.51
	Dissatisfied		Ref.				
	**Social support**	0.056	1.34	0.99–1.80	0.039	1.49	1.02–2.17
Neglkerke = 0.307 − 2LL = 1603.775 Model X^2^ = 287.673 (.000)							

Referent “class 1: *Low gain class*.” OR = odds ration; CI = confidence interval.

**Table 5:** Multinomial logistic regression of characteristics according to female work–family balance pattern.

Variable			Female (*n* = 751)					
			Class 2: Moderate Gain Class			Class 3: High Gain Class		
			*p*	OR	95% CI	*p*	OR	95% CI
**Household and** **couple characteristics**	**Number of children**	0.111		1.23	0.95–1.59	0.501	1.17	0.75–1.82
**Health** **characteristics**	**Subjective health status**	0.438		1.11	0.85–1.44	0.360	1.22	0.80–1.86
**Psychosocial characteristics**	**Depression**							
	Severe	0.151		0.60	0.30–1.21	0.482	0.53	0.09–3.09
	Mild/moderate	0.097		0.68	0.43–1.07	0.941	0.97	0.42–2.22
	Normal			Ref.				
	**Subjective happiness**	0.236		1.16	0.91–1.49	0.001	2.06	1.33–3.20
	**Daily stress**	0.378		0.86	0.61–1.20	0.580	0.86	0.50–1.48
	**Life satisfaction**							
	Satisfied	0.593		1.22	0.59–2.49	0.785	0.81	0.17–3.82
	Neutral	0.868		0.95	0.51–1.76	0.721	0.76	0.17–3.37
	Dissatisfied			Ref.				
	**Social support**	0.081		1.31	0.97–1.77	<0.001	6.08	3.42–10.82
Neglkerke = 0.191 − 2LL = 1139.993 Model X^2^ = 121.061 (.000)								

Referent “class 1: Low gain class” OR = odds ration; CI = confidence interval.

## Data Availability

Not applicable.
